# Immunological microenvironment in the testis

**DOI:** 10.1002/rmb2.12293

**Published:** 2019-08-29

**Authors:** Ning Qu, Yuki Ogawa, Miyuki Kuramasu, Kenta Nagahori, Kou Sakabe, Masahiro Itoh

**Affiliations:** ^1^ Department of Anatomy Tokyo Medical University Tokyo Japan; ^2^ Department of Anatomy, Division of Basic Medical Science Tokai University School of Medicine Kanagawa Japan

**Keywords:** andrology, immunology, spermatogenesis

## Abstract

**Background:**

The testis is specific in that it produces haploid germ cells of which autoantigens newly appear long after the neonatal immune tolerance. Under normal condition, these autoantigens are protected by the blood‐testis barrier formed by Sertoli cells. Thus, the testis is an immunologically privileged site where haploid cells are protected from autoimmune attack.

**Methods:**

The immunological microenvironment in the testis was experimentally investigated using mice and rats.

**Main findings:**

Not only the blood‐testis barrier but also various immuno‐suppressive factors are involved in the immune‐privileged testis. Indeed, germ cells transplanted into the xenogeneic seminiferous tubules could proliferate and differentiate with no aid of artificial immunosuppression. On the other hand, autoimmune orchitis could be experimentally produced by various methods of immunization with syngeneic or xenogeneic germ cell antigens.

**Conclusion:**

Our results indicate that the testis is immunologically privileged but also immunologically fragile organ. Therefore, the dual nature is critical for immunoregulation of testicular function.

## 1 APPEARANCE OF SPERMATIDS AND SPERMATOZOA AFTER THE ESTABLISHMENT OF IMMUNE TOLERANCE

**Figure 1:**
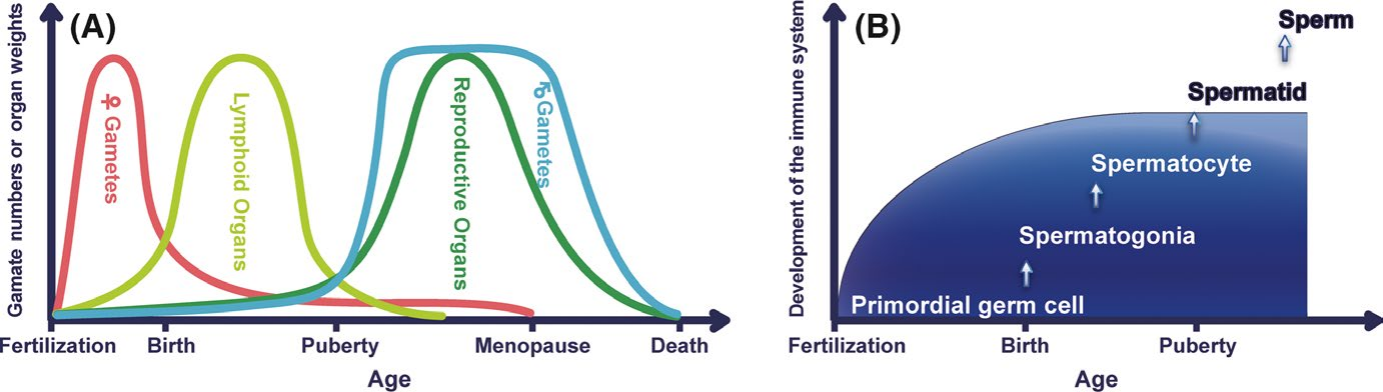
Chronological development of reproductive and immune systems. (A) The developmental relation among gametes, lymphoid organs, and reproductive organs. (B) Differentiation of male haploid germ cells after the establishment of immune tolerance

The reproductive system has evolved to allow self to interact with non‐self, whereas immune system has evolved to make distinction from non‐self to self, thereby allowing the emission of non‐self. It is well known that the life cycles of germ cells between males and females are completely different from each other. Primordial germ cells of both males and females are ready in the form of immature cells inside the 8‐week fetal gonads in humans. In 5‐month fetus, the number of primordial follicles in the ovaries reaches a peak approximately 7 million. Thereafter, the cell number decreases to approximately 2 million at the time of birth (Figure 1A). By puberty, number of primordial follicles decreases to several tens of thousands and then continues to decrease, disappear completely until the time of menopause at approximately 50 years old. On the other hand, in males, only a small number of spermatogonia develop within the testes from fetal to pre‐pubertal period; however, once puberty is reached, active spermatogenesis begins, and approximately 100 million spermatozoa are then produced, transported, and excreted on daily basis until gerontic period 1 (Figure 1A).

The developmental phase is different between lymphoid and gonadal tissues. It is well known that the lymphoid organs are the earliest to degenerate and develop in the embryo, but the reproductive organs are the slowest to mature (Figure 1A). The seminiferous tubules of mice contain spermatogonia at the day of birth, preleptotene spermatocytes a 1 week of age, pachytene spermatocytes at 2 weeks of age, round and oval spermatids at 3 weeks of age, the elongating spermatids at 4 weeks of age, and most mature (elongated) spermatid at 5 weeks of age.^2^ Spermatids and spermatozoa do not appear in the testis until puberty (starts at around 35 days of postnatal age) that far later than the period of neonatal immune tolerance from fetal to infant period. Therefore, autoantigens of these haploid cells may be targeted for immunological elimination (Figure 1B). Different from spermatogonia and preleptotene spermatocytes having 46 chromosomes, spermatids and spermatozoa emerging from meiosis have only 23 chromosomes but express various new differentiation autoantigens.^3^ Thus, the male reproductive organs have larger amounts of new autoantigens than those in the female ones.

## 2 TESTICULAR IMMUNO‐ENVIRONMENT FOR GERM CELL DIFFERENTIATION

**Figure 2:**
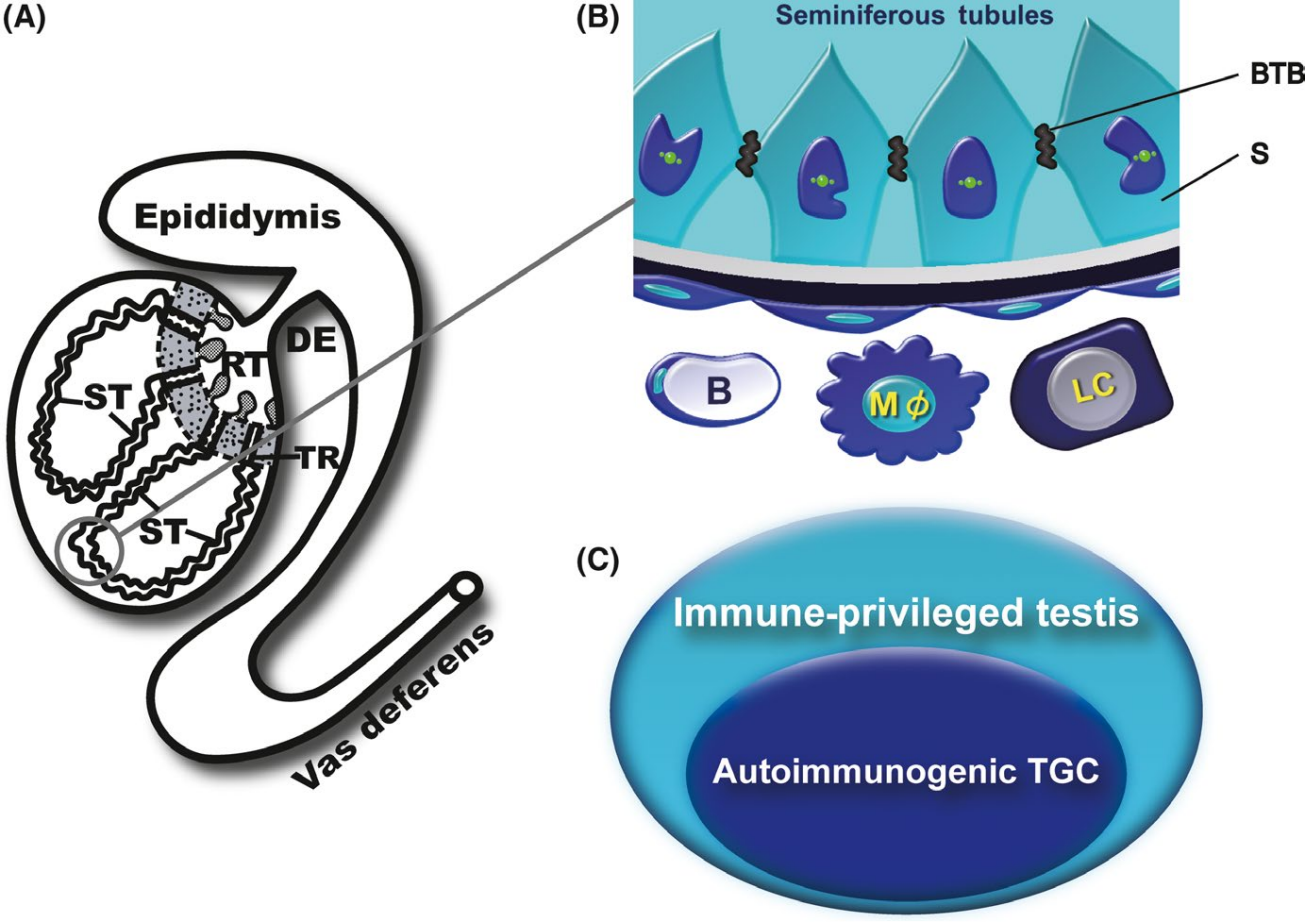
Micro‐circumstance for spermatogenesis. (A) Gross appearance of male reproductive organs. DE, ductuli efferentes; RT, rete testis; ST, seminiferous tubule; TR, tubuli recti. (B) Physiological protection of autoimmunogenic testicular germ cells. B, blood capillary; BTB, blood‐testis barrier; LC, Leydig cell; Mφ, macrophage; S, Sertoli cell. (C) Immuno‐environment for the protection of testicular germ cells

Spermatogenesis takes place within the convoluted seminiferous tubules, which then connect the tubuli recti (TR) and terminate at the rete testis (RT) (Figure 2A). Testicular germ cells (TGC) then leave the rete testis and are transported to the ductuli efferentes, epididymal ducts, and vas deferens (Figure 2A). Some studies show the development of testis in mice during the postnatal period. The tight junction between Sertoli cells develops between 10 and 16 days of age in the mouse and the presence of Sertoli cell junctions and seminiferous tubule lumen at 18 days of age.^4^, ^5^ Lee et al demonstrate that the area of RT can be detected at 10 days of age and the RT areas are significantly increased from 18 days of age.^6^ Autoimmunogenic spermatids and spermatozoa are believed to be protected from detrimental immune attacks by blood‐testis barrier (BTB), which is located at the base of the seminiferous tubules (Figure 2B). The BTB, established at 15 days of age in mice, is mainly composed of inter‐Sertoli cell junctions including tight junction, basal ectoplasmic specializations, gap junctions, and desmosome‐like junctions.^7^, ^8^ It is well known that the BTB protects post‐meiotic germ cells and is essential for the spermatogenesis. Recently, transplantation of rat spermatogenesis was demonstrated inside the BTB of immune competent mice.^9^ This indicates that the BTB protects not only autologous but also xenogeneic TGC from immunological elimination.

However, based on the findings of some researches, spermatids and spermatozoa are not completely isolated from immune system by the BTB in mammals; instead, these cells are not normally rejected by the individual's own immune system because of being maintained in a fine and subtle state of immune balance.^10^, ^11^, ^12^, ^13^, ^14^, ^15^, ^16^ All testicular cells, involving TGC, Sertoli cells, Leydig cells, testicular macrophages, peritubular myoid cells, endothelia of blood, and lymph capillaries and lymphocytes may modulate local immunity in the testis.^17^, ^18^, ^19^ These multiplex immuno‐modulation factors, involving various local elements of different origins, participate in the formation of an immunologically privileged testis (Figure 2C).

**Figure 3:**
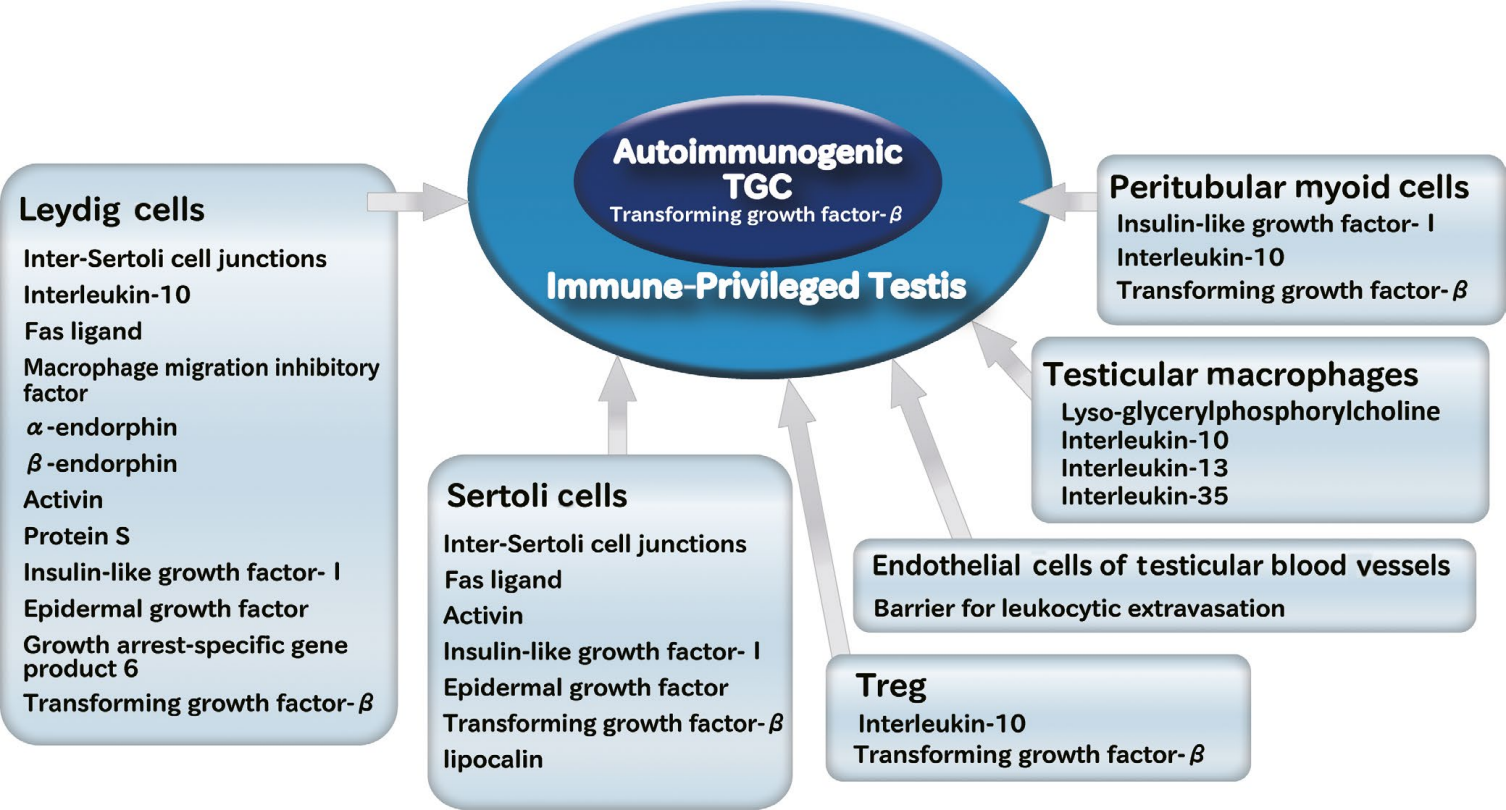
Various immuno‐suppressive factors in the testis

Besides the function of BTB as an immunologic barrier, numerous immunoregulatory molecules including androgens, macrophage migration inhibitory factor, activin, Fas ligand, IL‐10, IL‐35, transforming growth factor‐beta, programmed death‐ligand 1, Toll‐like receptors, and TAM (Tyro‐3, Axl, and Mer) receptors were secreted by the testicular cells. These molecules play critical roles in regulating immune responses in the testis (Figure 3).^20^, ^21^, ^22^, ^23^, ^24^, ^25^, ^26^, ^27^ Although the actual contribution and combination of each factor are still unclear, a wide variety of mechanisms is estimated to be effective for inhibition of autoimmune responses in the testes.

## 3 BREAKDOWN OF TESTICULAR IMMUNE PRIVILEGE RESULTS IN TESTICULAR AUTOIMMUNITY

At the puberty, the male immune system first perceives intolerant haploid cell antigens in the testis as completely different from any other previously encountered and already tolerated diploid cell antigens. In particular, spermatids, which have more abundant cytoplasm than spermatozoa, should contain various autoimmunogenic antigens of large amounts. Therefore, the testes in which spermatids emerge from miosis may be more sensitive to autoimmune inflammation than the vas deferens and epididymis in which mature spermatozoa but not spermatids are compacted.

If the testicular immune privilege is upset under some condition, immune responses against the TGC autoantigens should be induced. The characteristics of testicular autoimmunity include the detection of inflammatory cell infiltration into the testis, disturbed spermatogenesis, testicular antigens‐specific T‐cell response, the specific serum autoantibodies, and binding of the autoantibodies and complements in the testis,^28^ but it is clinically difficult to detect all these features in men.

Some reports have demonstrated the lymphocytic infiltration and immune deposits in specimens of testicular biopsies from infertile men.^29^, ^30^, ^31^, ^32^, ^33^, ^34^, ^35^, ^36^, ^37^ The characteristic feature is spermatogenic disturbance surrounded by lymphocytic infiltration. Testicular lymphocytes increased in infertile patients with sperm‐autoimmunity, and the predominance of CD8 + T cells was demonstrated in the testis interstitium.^38^, ^39^ Furthermore, diagnostic testicular biopsy declared the significant presence of CD68 + macrophages in the testes of all infertile patients with maturation arrest, Sertoli cell‐only syndrome, mixed atrophy syndrome, and idiopathic infertility showing normal spermatogenesis.^40^ These macrophages were located in the testicular interstitium and in/around the seminiferous tubules and expressed the genes of IL‐1 and TNF‐α. The phenotypical characterization of testicular leukocytes demonstrated that cell counts of all examined populations (T cells, B cells, macrophages, and mast cells) increased in Sertoli cell‐only syndrome and maturation arrest when compared with those in normal spermatogenesis.^41^

The increase in testicular mast cells closely contacted to the seminiferous tubules indicates a relationship between mast cell proliferation and the BTB dysfunction.^42^ Testicular germ cells autoantigens leak beyond the BTB when the BTB is functionally damaged. This leads to a continuous supply of the autoantigens to the immune system, with the resultant chronic inflammation in the testis for a prolonged spermatogenic disturbance.^19^, ^43^ Particularly, the BTB is demonstrated to be incomplete at the TR and the RT.^44^, ^45^ This implies that the testicular tissue around the TR is a site where autoreactive lymphocytes can gain access to autoimmunogenic TGC antigens. Furthermore, in rodent study, it was found that many macrophages accumulate around the TR and a few of them penetrate into the TR.^17^ Under normal condition, they may take the materials leaked from the TR to inhibit the induction of inflammatory responses.^46^, ^47^ However, when the testicular immune privilege becomes unstable and upset, the TR should be immunological‐specific region, where lymphocytes are attracted. Because diagnostic biopsy of the TR and the RT is clinically impossible, therefore, it is quite difficult to know the histological appearance in infertile patients.^19^, ^48^ That is why the clinical data on the relation between autoimmune orchitis and male infertility have been still unclear.

On the other hand, as a model of acquired idiopathic spermatogenetic failure, research on experimental autoimmune orchitis (EAO) has been widely conducted in mice, rats, guinea pigs, and other animals (Table 1). Conventionally, EAO was induced by subcutaneous injection of testicular antigen mixed with complete Freund's adjuvant (CFA) containing killed *Mycobacterium tuberculosis* in guinea pigs and by subcutaneous injection of testicular antigen with CFA and killed *Bordetella pertussis* in mice.^49^, ^50^, ^51^ Later, Itoh et al 11, 12, 13 reported that EAO could be easily and simply induced in mice by only subcutaneous injection of syngeneic TGC with no adjuvant. In experimental animals with unilateral testicular trauma, Naito et al 52 also succeeded in inducing sympathetic EAO, EAO in the contralateral testis, with no immuno‐potentiating drugs.

**Table 1:** Various methods for the induction of experimental autoimmune orchitis

Various methods for the induction of EAO
① Immunization with testicular antigens alone
Immunization with syngeneic TGC
Immunization with allogeneic TGC
Immunization with xenogeneic TGC
Multiple immunization with testicular homogenate
Abdominal placement of donor testes
② Immunization with testicular antigens and immuno‐potentiating agents
Treatment with cyclophosphamide and the following immunization with TGC
Immunization with testicular antigens or homogenate emulsified in CFA
Immunization with mixture containing testicular homogenate and BP
Immunization with testicular antigens or homogenate emulsified in CFA and the following intravenous administration of BP
Immunization with testicular antigens and *Klebsiella* lipopolysaccharide
③ Local injury of the testis
Traumatic injury of unilateral testis
Intrinsic disorder in the testis (abnormal spermatogenesis, abnormal TGC clearance, abnormal BTB)

Abbreviations: BP, Bordetella pertussis antigens; BTB, blood‐testis barrier; CFA, complete Freund's adjuvant; TGC, testicular germ cells.

As EAO can be induced by only exposing TGC to the immune system outside the BTB without any artificial immune enhancement,^11^ it is noted that this disease model is closer to the clinical cases, in which focal injuries in the testis such as ischemia and trauma damage the seminiferous tubules, followed by leakage of the TGC to the outside of the tubules. Furthermore, the area of predilection of lymphocytic infiltration is in and near the interstitial tissue adjacent to the TR and the RT in both the human cadavers and the mouse EAO model.^43^, ^52^ Therefore, even if no inflammatory lesion is found on biopsy at some testis regions far from the TR and RT of infertile men, a possibility of inflammation involving the mediastinum testis remains.

It is clinically general that the presence of anti‐sperm antibodies is a key for diagnosis of male infertility of immunologic origin,^53^ and measurement and analysis of anti‐sperm antibodies have been extensive.^54^ Moreover, autoantibodies against the other testicular cells and components such as Sertoli cells, Leydig cells, and basement membrane of the seminiferous tubules were also detected in male infertility.^55^, ^56^ However, it is insufficient to diagnose testicular autoimmunity only with detection of the autoantibodies on sera and/or semen. The role of autoantibodies in EAO induction still remains obscure. Active EAO is induced by immunization with testicular antigens, and passive EAO is inducible by transfer of testis‐specific lymphocytes. Therefore, both cellular and humoral immune responses are induced by immunization for active EAO while cellular but not humoral immunity is critical for passive EAO. Indeed, the histopathologic patterns of the initiation of inflammation in active and passive EAO differ from each other.^13^, ^57^, ^58^ This indicates that EAO is generally CD4 + T‐cell dependent, but B cells, plasma cells, and their production of autoantibodies should affect the inflammatory pattern of EAO.

## 4 HOW THE TESTICULAR IMMUNE PRIVILEGE AGAINST XENOGENEIC GERM CELLS IS EFFECTIVE OR BROKEN?

**Figure 4:**
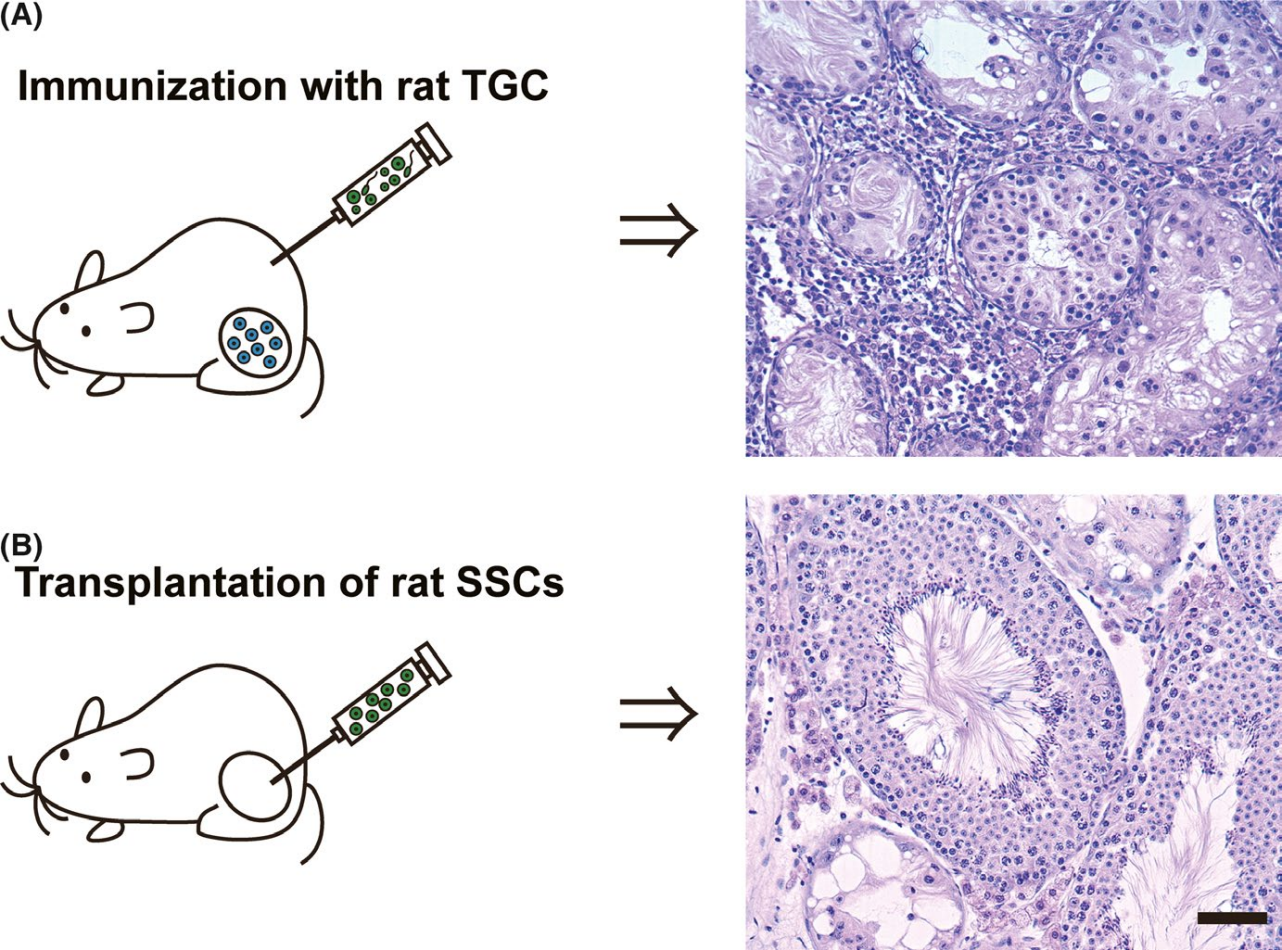
Testicular tissue responses against xenogeneic germ cells in immunocompetent mice. (A) Immunizations with xenogeneic Testicular germ cells (TGC) alone. Note the inflammatory cell infiltration in the thickened intertubular interstitium and devastation of seminiferous tubules leading to hypospermatogenesis in the testes. (B) Xenogeneic spermatogonial stem cells (SSCs) transplantation into endogenous TGC‐depleted testes. Note the concurrent presence of normal‐appearing spermatogenesis and aspermatogenesis in the testes. Bar = 20μm

Previously, it has been reported that delayed type hypersensitivity (DTH) against allogeneic or xenogeneic TGC was elicited in syngeneic TGC‐immunized mice 59 and the following studies revealed that DTH rather than humoral immunity against TGC antigens is critical for TGC‐induced EAO induction.^12^, ^14^, ^60^, ^61^, ^62^ Recently, it was found that immunizations with rat TGC alone can induce murine EAO without using adjuvants 63 (Table 1; Figure 4A). The DTH against murine TGC was significantly elevated in mice immunized with syngeneic or xenogeneic TGC (rat TGC). Serum autoantibodies to murine TGC determined by enzyme‐linked immunosorbent assay were significantly elevated in the same manner as the results of DTH. The reactions of immune sera with frozen sections of normal murine seminiferous tubules indicate that the interspecies common antigens of immature TGC rather than mature ones are important for the induction of EAO.^63^

On the other hands, it has been known that rat spermatogenesis can occur in the seminiferous tubules of congenitally immunodeficient recipient mice after transplantation of rat spermatogonial stem cells (SSCs).^64^ Experimentally immunosuppressed adult mice were found to be also useful as the recipients. When hamster SSCs were transplanted into the testes of infant rats with immature immune system, hamster spermatogenesis could be detected within the rat seminiferous tubules.^65^ Later, transplantation of rat SSCs into immunocompetent mice was investigated. The results showed that transplanted rat SSCs could undergo complete spermatogenesis in recipient mouse testes, and many rat spermatozoa could be detected in the recipient epididymides 9 (Figure 4B). This implies that transplanted rat spermatogonia could undergo complete spermatogenesis in normal immune system of the recipient mice.^66^ Therefore, xenogeneic rat germ cells can be immunologically segregated and supported in the recipient's seminiferous tubules formed by Sertoli cells, basal lamina, and peritubular myoid cells of immunocompetent mice. In spite of the high immunogenicity of xenogeneic TGC, they can be remained within the seminiferous tubules, epididymal duct, and vas deferens without eliciting inflammatory reactions.

Furthermore, to investigate testicular immune privilege more clearly, transplantation of testicular tissue fragments or whole donor testis surgical anastomosis with recipient's blood vessels was tried. It became evident that intratesticular transplantation of testicular tissue fragments from immature donors in several species (mouse, rabbit, cat, dog, monkey, and gazelle) into immunodeficient recipient mice resulted complete spermatogenesis.^67^, ^68^, ^69^, ^70^, ^71^, ^72^, ^73^ Concurrently, testis with testicular artery, testicular vein, ductuli efferentes, epididymides, and a part of vas deferens are transplanted into syngeneic male, allogeneic male, xenogeneic male, syngeneic male castrated at various ages, and syngeneic females, for studying testicular immunology from different aspects.^74^, ^75^, ^76^, ^77^, ^78^, ^79^, ^80^ If the immune privilege status in the transplanted organs is broken down in recipients, EAO‐like inflammatory lesions may be induced in the transplanted testes and epididymides. Historically, Lee et al 74 are the first to try transplantation of allogeneic testis in the rat. In the transplanted allogeneic testis, lymphocytes and macrophages were detected at perivascular region, but none infiltrated into the seminiferous tubules on day 3. Necrosis of seminiferous epithelium first appeared on day 3 and became progressive thereafter. During the first 7 days, perivascular inflammatory cell infiltration was also found in the epididymis. The epididymal changes included infiltrates of mononuclear and polymorphonuclear cells, abscess formation, fibrosis, and granuloma formation. Because the technical difficulty for testis‐transplantation, the further study is inadequacy. It is important to investigate the transplant immunology of the testis to determine how grafts of testicular cells, tissues, or whole organ are immunologically accepted or rejected in “immunocompetent” recipients.

## 5 CONCLUSION

Spermatogenesis is the process by which spermatozoa develop from SSCs through mitosis and meiosis. It starts at puberty and usually continues uninterrupted over the reproductive lifetime. Spermatids and spermatozoa are the lastly appearing cells in individual mammal, and they have various developing antigens that are unfamiliar with self‐immune system. Various EAO models can be easily produced without using adjuvants in both immunocompetent and immunodeficient animals. This ease of disease induction is far different from other experimental organ‐specific autoimmune diseases. Therefore, although the testis is regarded as an immunologically privileged organ and resistant to inflammatory responses, it is also highly susceptible to autoimmune inflammation. Normal spermatogenesis appears to be dependent on the sheltered microenvironment for TGC and also on a fine balance between effective and suppressive immunity against physiologically leaked TGC antigens in “natural autoimmunity.”

This article does not contain any studies with human subjects performed by the authors. All the experimental protocols in this study were carried out in accordance with the guidelines of the National Institutes of Health and were approved by the Tokyo Medical University Animal Committee.

## CONFLICT OF INTEREST

All authors have no conflicts of interest to declare.
